# Reply to “Comment on Effectiveness of a Group B Outer Membrane Vesicle Meningococcal Vaccine in Preventing Hospitalization from Gonorrhea in New Zealand: A Retrospective Cohort Study, *Vaccines*, 2019, *1*, 5; doi:10.3390/vaccines7010005”

**DOI:** 10.3390/vaccines7010032

**Published:** 2019-03-15

**Authors:** Janine Paynter, Felicity Goodyear-Smith, Jane Morgan, Peter Saxton, Steven Black, Helen Petousis-Harris

**Affiliations:** 1 Department of General Practice and Primary Health, University of Auckland, Auckland 1142, New Zealand; f.goodyear-smith@auckland.ac.nz; 2 Sexual Health Services Waikato District Health Board and Honorary Senior Lecturer, School of Medicine, University of Auckland, Auckland 1142, New Zealand; Jane.Morgan@waikatodhb.health.nz; 3 Department of Social and Community Health, University of Auckland, Auckland 1142, New Zealand; p.saxton@auckland.ac.nz; 4 Department of Pediatrics, Cincinnati Children’s Hospital, Cincinnati, OH 45229-3039, USA; stevblack@gmail.com; 5 Immunisation Advisory Centre, Department of General Practice and Primary Health Care, University of Auckland, Auckland 1142, New Zealand; h.petousis-harris@auckland.ac.nz

Kenyon (2019) is correct to suggest that there is limited evidence of longer term effectiveness. However caution is also warranted on making a firm conclusion that the vaccine has no longer term effectiveness because the evidence for this is also limited. There was a lack of power in both studies, due to at least two factors, to examine a longer term effect. This is different from an effect not being there. These factors were (1) a much smaller proportion of younger, unvaccinated individuals, and (2) many of younger vaccinated individuals within the cohort would not have been at risk yet because at the time of the study they were unlikely to be sexually active. The effect of a booster has also not been considered. It may be worthwhile revisiting the data in a few years to look again at this younger cohort.

Finally, risk of gonorrhoea for most people declines dramatically once they reach 30 years of age, so a lifetime of vaccine effectiveness is less important for population management of gonorrhoea compared to other infectious disease such as influenza or pneumonia. 

